# Common Strategies and Factors Affecting Off-Line Breath Sampling and Volatile Organic Compounds Analysis Using Thermal Desorption-Gas Chromatography-Mass Spectrometry (TD-GC-MS)

**DOI:** 10.3390/metabo13010008

**Published:** 2022-12-21

**Authors:** Kinga Westphal, Danuta Dudzik, Małgorzata Waszczuk-Jankowska, Beata Graff, Krzysztof Narkiewicz, Michał Jan Markuszewski

**Affiliations:** 1 Department of Hypertension and Diabetology, Medical University of Gdansk, 80-214 Gdansk, Poland; 2 Department of Biopharmaceutics and Pharmacodynamics, Faculty of Pharmacy, Medical University of Gdansk, 80-416 Gdansk, Poland

**Keywords:** metabolomics, mass spectrometry, volatile organic compounds (VOC), thermal desorption, gas chromatography, breath sampling and analysis

## Abstract

An analysis of exhaled breath enables specialists to noninvasively monitor biochemical processes and to determine any pathological state in the human body. Breath analysis holds the greatest potential to remold and personalize diagnostics; however, it requires a multidisciplinary approach and collaboration of many specialists. Despite the fact that breath is considered to be a less complex matrix than blood, it is not commonly used as a diagnostic and prognostic tool for early detection of disordered conditions due to its problematic sampling, analysis, and storage. This review is intended to determine, standardize, and marshal experimental strategies for successful, reliable, and especially, reproducible breath analysis

## 1. Introduction

Human breath is composed of inorganic compounds, such as nitrogen, oxygen, carbon dioxide, argon, and water vapor, and contains trace volatile organic compounds (VOCs) [1]. VOCs represent the physiological processes that take place in the body and a detailed analysis of their levels and identities provides information about overall health conditions and prospective forms of illness [2]. These volatile molecules are endogenously generated in the body and also may be absorbed from the environment. In order to determine any pathological process in the body or to monitor metabolism, only endogenous molecules should be determined. Endogenously generated VOCs circulate in the blood, cross the blood–air barrier, and occur in exhaled breath at trace concentrations (parts-per-million by volume (ppmv) and parts-per-billion by volume (ppbv) levels or lower) [3].

Breath analysis has been used as a painless diagnostic tool since ancient Greece. It is known that some diseases trigger a specific breath odor, such as urine-like or “rotten apple” smells which are typical for kidney diseases and diabetes, respectively [4]. In 1970, Pauling et al. determined over 200 components in human breath, and thereby pioneered the modern breath analysis [5]. Since then, interest in breath monitoring for disease diagnosis has continually increased; however, this method is still not commonly used in clinical practice [6]. This is because, in principle, technical and methodological issues limit the reproducibility and reliability of the obtained results. The main obstacles which should be overcome are as follows: (i) inadequate breath sampling, (ii) non-dedicated storage and preconcentration methods, (iii) unsuitable desorption and analysis, (iv) exogenous contamination, and (v) misuse of computational tools [7]. 

There are two types of metabolome analysis: targeted and nontargeted metabolomics. Targeted metabolomics is used to determine abundancies and concentrations of expected metabolites, while nontargeted metabolomics constitutes a global analysis of all the measurable analytes [8]. The main goal of successful breathomics (breath metabolomics) is to determine the compounds (biomarkers) whose concentrations are significantly different from healthy controls [9]. Moreover, a clinically useful biomarker should reflect a particular pathological state and its level fluctuates only for this disease process [10]. Many research groups have performed studies to determine biomarkers in breath, and these biomarkers have been used in the diagnosis and monitoring of such diseases as asthma, hemolysis, different types of cancer, cardiovascular heart diseases and also viral infections [11,12,13,14,15,16]. One should keep in mind that many VOCs that are present in human breath characterize the physiological state of an individual. In breathomics, it is essential to distinguish healthy patients from those with abnormal metabolisms.

Breath analyses have interested mankind for centuries, since by only using the sense of smell it has been possible to detect inorganic gases such as ammonia and hydrogen sulphide that diagnose various diseases due to impaired liver and kidney function. With the development of analytical techniques, the identification of breath components that could be indicative of any pathological state in the human body finally became possible. Nowadays, advanced analytical tools have enabled monitoring of human health, as well as early detection or prediction of any pathological state. Regular examination of human health via exhaled breath analysis has a significant meaning with regard to the prevention and detection of several diseases and also a reduction in total healthcare costs and an improvement in life expectancy. 

This review is intended to describe a chosen off-line variant of human breath analysis that employs Tedlar bags and thermal desorption to develop its potential and application in the diagnosis of various diseases in the future. We discuss all the experimental challenges that could be encountered, as well as determine recommendations that should be considered before, during, and after off-line breath sampling and analysis. 

## 2. Patient-Related Presampling Recommendations

### 2.1. Diet

Clinically relevant metabolites have endogenous origins and their levels in exhaled breath can be altered by several factors, for example, (i) exogenous VOCs absorbed from ambient air; (ii) gender, genetics, habits (smoking and diet), medications, age; (iii) sampling; (iv) lung physiology; (v) hypo- and hyperventilation; and (vi) physical-chemical properties of VOCs diffusing from blood to air, etc. [1,17]. To perform reliable identification of breath constituents, presampling preparations should remain an indispensable part of the breath sampling procedure. The general principles that must be strictly adhered to in order to avoid false-positive and false-negative results include: (i) fasting for a minimum of 30 min. to overnight before sampling; (ii) resting in a sampling room for 5 to 15 min. and (iii) avoiding perfumed cosmetics, garlic, onion or any spicy food and flavored drinks. If the need arises, drinking only unflavored water is allowed [18,19,20,21,22,23]. Eating habits are individual traits of any person and affect the VOCs in exhaled breath; thus, abstaining from eating a particular food and fasting are especially important in the analysis of exhaled breath. Raninen et al. [24] determined a postprandial decrease in 2-methylbutyric acid and increases in ethanol, 1-propanol, acetoin, and propionic acid concentrations in breath samples taken from patients eating high-fiber foods. Meal frequency also poses a symptomatic feature that indicates the current status of digestion—a variable which especially should be heeded in an untargeted breath analysis. Barańska et al. [25] determined the different VOCs in breath samples taken from patients who, for the first four weeks, maintained a gluten-free diet, and then switched to a normal diet. Seven compounds that distinguished samples were qualitatively determined: 2-butanol, octane, 2-propyl-1-pentanol, nonanal, dihydro-4-methyl-2 (3H)-furanone, nonanoic acid, and dodecanal. Moreover, the results revealed that these metabolites were temporarily excreted, which suggested that a gluten-free diet only reversibly modified the quality of the exhaled air. It is also worth noting that volatile compounds can result from microbial metabolism and can be easily detected in exhaled breath. During the microbial fermentation process of non-digestible food ingredients, many VOCs are produced, including short-chain fatty acids [26].

### 2.2. Breathing Manners

Presampling recommendations also refer to breathing manners, such as breath-holding and forceful exhalation, which are factors that alter the levels of various VOCs. Increases in the content of pentane, isoprene, 2-propanol, acetaldehyde, acetone, methanol, and dimethyl sulphide were determined in breath samples taken from patients who held their breath before exhalation. The elevated levels of these compounds were directly related to their prolonged diffusion from the alveoli to the airway [27]. Similarly, the levels of various VOCs can be affected by the expiratory flow rate (EFR). However, in that case, several contradictory results concerning the influence of EFR on the levels of various VOCs in human exhaled breath exist. According to Doran et al. [19], a higher EFR could reduce the concentration of acetone and phenols, but in turn, Bikov et al. [28] did not determine any correlation between the EFR and acetone levels. In another study [29], it was confirmed that a higher flow rate significantly affected acetone concentration in exhaled breath samples. Since breathing mode can have a significant impact on the levels of VOCs, breath samples should be taken with the use of a device that does not cause a rise in airway resistance. For off-line breath analysis, it is recommended to use a mouthpiece with a diameter larger than 1 cm [30]. 

### 2.3. Age

Another factor that has a measurable impact on exhaled VOC levels is age. It has been determined that ammonia concentration was significantly higher in breath samples taken from elderly patients [31], while isoprene level was recognized to be lower in adults’ exhaled breath [32]. Diseases also modify the level of particular compounds in exhaled breath. Inorganic compounds such as nitric oxide and carbon monoxide can be detected in asthma and cardiovascular diseases, respectively [33], while elevated levels of markers indicating oxidative stress can be a hallmark of heart transplant rejection [34]. Moreover, disease-associated biomarkers sometimes may have a negative impact on breathing measurements in a particular experiment. As a consequence, participants that suffer from such a disease should be excluded from a study group before sample collection. 

### 2.4. External Fragrances and Smoking

Among all the factors that alter the composition of breath, the most important factor that must be supervised in breath analysis is smoking behavior [35]. Smoking modifies the VOC profile in the general population, and among other classifiers such as gender, age, and BMI, it significantly separates smoking from non-smoking patients. Elevated concentrations of the following compounds can be detected in the breath of smokers and passive smokers: acetonitrile, 2,5-dimethylfuran, benzene, toluene, pentane, ethane, and styrene [36]. Among them, acetonitrile is recognized as a hallmark of the smoking habit because its level is heightened for almost a week after cessation before it reaches a normal (non-smoking) concentration [37]. It should also be mentioned that any other air contaminants also enter the body through inhalation or skin and change the concentrations of VOCs detected in exhaled air. Air fresheners, household chemicals, and beauty products emit fragrances that can be easily detected during analysis [38]. However, it should be emphasized that it is almost impossible to exclude all these molecules that may have their origin in external fragrances from the composition of breath, nevertheless, it is worth paying attention to. 

In 2018, Hanna et al. [39] proposed the main factors that should be taken into consideration in investigating the role of VOCs in cancer diagnosis. In fact, all these factors may find application in any VOCs analysis and they include:

(i) Patient-related factors, i.e., their physiological conditions and clinical confounding factors such as medication;
(ii) Environmental considerations, i.e., background air measurement, and determination of contaminant VOCs originating from sampling devices.

Breath sampling continues to be an advantageous process that requires an understanding of exhalation physiology and interfering factors. Despite the fact that exhaled air is a less complex matrix than blood and non-invasive sampling procedures are not stressful for patients, background VOC levels produced by regular metabolic processes and derived from environmental exposure still remain an issue. To obtain statistically significant results, it is important to properly match patients in the study with a control group. 

Defining a proper reference control group is a recognized challenge in clinical studies. In the case of breath analysis, similar environmental factors should be particularly considered. One of the possible options is the recruitment of a person, for example, a partner or another relative from the immediate surroundings of a patient. In such circumstances, many associated variables such as habits or diet reduce the risk of unwanted variables and bias at an early stage of the experiment.

## 3. Off-Line Breath Sampling Devices

### Tedlar Bags as Off-Line Sample Containers

There are several types of containers which can be used for off-line breath sampling, such as (i) bags [40], (ii) glass and metal canisters [41], (iii) BioVOC [42], (iv) RTubeVOC [43], (v) SOFIA [44], and (viii) ReCIVA [45]. Among them, bags made up of polyvinyl fluoride (PVF) film (commercially available as Tedlar bags) are the most frequently used for gas analysis application [46]. In breathomics, there are two types of commonly employed Tedlar bags: (i) transparent and (ii) black-layered, in which polymeric film contains carbon additive to protect the sample from UV light [40]. For clinical purposes, the ideal breath container must meet the following fundamental requirements: be reusable, impermeable, and should ensure proper storage of sample components [47]. Because Tedlar bags are popular sampling receptacles, they can have common disadvantages. There are several bag-related issues that alter exhaled breath sampling, such as (i) background volatiles, (ii) sample volume (single vs. multiple breaths), (iii) expiratory flow rate, and (iv) external factors (humidity and temperature). In order to exploit the full potential of Tedlar bags in breath analysis, it is essential to use, handle, and store them with care.

To avoid background and external contamination, it is necessary to determine effective cleaning guidelines. New and used bags should both be cleaned before each use. Gaseous pollutants can be easily removed from sampling bags via continuous flushing with clean air. However, the cleaning protocol for contaminants adsorbed onto the bag walls must be conducted in a more rigorous manner [48]. According to Steegs et al. [40], bags should be equipped with two valves that enable their continuous flushing with clean air. They recommend heating bags to 60 °C and applying a clean gas flow with the rate of 25 L/h for 2 h. Similarly, Grabowska-Polanowska et al. [49], in order to remove contaminants, rinsed the bag with synthetic air, filled the bag with the same agent, and heated it at 60 °C for 12 h. In turn, Mochalski et al. [50] flushed a 3 L Tedlar bag with high-purity nitrogen five times. To minimize the background, bags can also be rinsed three times with ultrapure air, and then conditioned at 50 °C, which should be followed by flushing with another 3 L of ultrapure air [51]. Moreover, McGarvey and Shorten [52] repeated five cycles of filling a bag with high-purity nitrogen and gradually increased the temperature to 27 °C during the process. An alternative and more rigorous method was proposed by Beauchamp et al. [48]. Filling a bag with zero air and equilibration was repeated three times, followed by O/N bake-out at 95 °C. Examples of cleaning guidelines are listed below in Table 1.

Although according to the manufacturers Tedlar bags are disposable, due to economic reasons, they are commonly reused. Hence, it has been necessary to determine the effectiveness of cleaning guidelines. Mochalski et al. [40] measured the concentrations of a standard VOC mixture (i) after seven days of storage and before cleaning as well as (ii) after the execution of the following cleaning protocol. The cleaning protocol included: (i) five-fold flushing with high purity nitrogen, (ii) bag’s conditioning with ultrapure nitrogen at 50 °C for 12 h, (iii) five-fold flushing with high purity nitrogen, and (iv) conditioning of the bag filled with nitrogen at RT for 12 h. To determine cleaning efficacy, the concentrations of a standard VOC mixture were measured before and after cleaning and the results revealed that, among 41 standard species, only four species were detected in a purified Tedlar bag. It should be mentioned that this cleaning procedure appeared to be invasive. One should keep in mind that the reusability of sampling bags also leads to their ageing and causes damage due to mechanical stress, which modifies the structure of the polymer film. In general, Tedlar bags are fragile and their reuse should be limited.

It is also recommended to stabilize sampling bags before the preconcentration step, usually for 1 h at 37 °C, which prevents water condensation on the walls of the bags [53]. However, it should be noted that the elevated temperature counteracts sample condensation and absorption to the inner film which promotes its diffusion. Moreover, it has been determined that methanol, a commonly used standard solvent, interacts with polyvinyl film via hydrogen bonds and despite a relatively high vapour pressure, it easily and irreversibly adsorbs on the polymer film [52].

As previously mentioned, Tedlar bags are one of the containers that could be successfully employed in a VOC analysis. Although such an approach belongs to the classical methods of breath analysis, it does not require employing trained manpower to collect breath samples. Moreover, Tedlar bags belong to rather affordable breath containers and can be easily cleaned which provides the possibility of their reuse. As compared with other sampling devices, Tedlar bags significantly reduce the costs of an analysis, which also confirms their untapped potential and the necessity to standardize the method of breath screening that employs such sample containers.

## 4. Exhaled Breath Sampling Procedure

### 4.1. Breath Fractions in the Respiratory Track

Human breath can be divided into three main fractions: (i) late expiratory, (ii) mixed expiratory, and (iii) end-tidal (alveolar) air [54]. Mixed expiratory air constitutes the total exhaled breath, including dead-space air, that does not participate in blood gas exchange and contains both endogenous and exogenous VOCs [55]. Late expiratory air represents dead-space air and the end part of the breath cycle; so far, a standard practice for collecting this breath fraction has not been developed. In turn, end-tidal air contains the highest concentration of endogenous compounds and also represents the internal level of exogenous compounds providing important metabolomics information [56]. Alveolar air sampling requires the subject to take a deep breath before filling the bag. In this case, the first 150 mL of the breath is regarded as a contaminant and usually is rejected [57]. In turn, dead-space air sampling does not require the subject to breathe because the breath is collected with a pump which transfers exhaled air from the mouth to the bag [58]. From a clinical perspective, breath can be subdivided into (i) gaseous breath (GB), (ii) volatile breath (VC), and (iii) breath condensate (BC). Novel breath tests usually employ VC and BC; however, GB fraction is also significant because it contains nitric oxide, which is a biomarker of eosinophilic airway inflammation and its level is measured with the use of a clinically approved FeNO breath test [59]. Despite the fact that the VC fraction constitutes less than 1% of the total usable volume of the lungs, it contains an infinite number of low molecular weight compounds that can be qualitatively and quantitatively measured with modern analytical tools, revealing their clinical utility [43]. Exhaled BC can be broken down into (i) the airway lining fluid-derived aerosolized droplets and (ii) water-soluble volatile gases that indicate any biochemical and inflammatory process in the lung [60]. This type of breath can be easily collected by cooling exhaled air [61].

### 4.2. Tedlar Bags for Exhaled Breath Sampling

Breath sampling with the use of Tedlar bags does not differentiate particular breath fractions. Despite this, the contribution of dead-space air to the total volume of breath is rather low and does not reduce sensitivity [62]. The typical breath collecting kit consists of (a) a mouthpiece and (b) a sampling bag. In some cases, a mouthpiece and a bag are separated by a hydroscopic unit containing silica gel. Then, a mouthpiece is fitted with an antisyphon valve that hinders inhalation of hygroscopic agent. It should be noted that the hydroscopic unit is not commonly employed because silica gel can interact with several breath compounds [43]. All sampling methods require standardization and the choice of methodology depends on the application. It is possible to successfully collect breath samples from patients of different ages and disease states, however, it requires some adjustments to be applied. For example, in the case of children under the age of 4 years, it is more convenient to take breath samples with the use of a mask instead of a mouthpiece. Because this collection technique includes air derived from both nasal and oral cavities, samples cannot be compared with breath collected from a similar group using a mouthpiece. Similarly, if the sample is taken during mechanical ventilation, it does not contain air from the upper respiratory tract. As a consequence, such samples can only be compared with those taken in exactly the same way [43]. Exemplary clinical applications of breath assays conducted in a patient population with particular age and disease, as well as breath sampling details, are compiled in Table 2.

It is recommended to sample larger volumes of breath instead of a single breath because such an approach guarantees higher reproducibility and lower variability [62]. To determine the levels of trace VOCs in exhaled breath, any contamination must be avoided and an appropriate sampling methodology must be followed. The standardized methods for off-line sampling still have not been defined, hence, sampling methods should be tailored to the needs of research.

## 5. Stability of VOCs in Tedlar Bags

### 5.1. Breath Sample Storage in Tedlar Bags

Sample integrity and storage can be influenced by several factors and remain an ultimate challenge in breathomics. Extremely low concentrations of reactive breath constituents favor external and background contamination, sample loss, and are prone to interactions between sample components. Some exogenous reactive species can interact with water vapor resulting in contaminant compounds. For example, the reaction between NO_2_ and water leads to the formation of light-sensitive nitrous acid (HNO_2_). This compound undergoes photodissociation and produces hydroxyl radical, which may oxidize compounds in breath samples [75]. Sample loss can also be associated with component adsorption [52]. Steeghs et al. [40] determined significant sample loss, which could have been the result of the sample sticking to the septum in the inlet valve. Four hours after sampling, from among seven standard compounds (methanol, acetaldehyde, acetone, isoprene, benzene, toluene, and styrene), the styrene concentration decreased by more than 15% and total sample loss ranged from 5% to 47%. In turn, Beauchamp et al. [48] performed an experiment in which concentration of 12 breath constituents in ppbv range was monitored over a 70 h storage period and determined that a sample should be analyzed within 10 h of sample collection to provide admissible recovery. The level of recovery from bags can also be affected by (i) the volume/surface area ratio; (ii) the chemical properties of the sample, and (iii) bag size. Mochalski et al. [50] performed a seven-day standard stability test to determine how surface-to-sample volume ratio (SA:V) influenced sample integrity. For that purpose, polymer bags were filled with different volumes of a 41-component standard mixture: 0.6 L, 1.2 L, and 2.4 L, which corresponded to 20, 40, and 80% of their total volume and monitored the concentration of standards at the following intervals: 0.16, 6, 24, 48, 72, 126, and 168 h after filling. The particular SA:V ratios are depicted in Table 3.

After 7 days of storage, a good standard mixture recovery was determined for the bag with an SA:V ratio equal to 73. Moreover, high molecular weight compounds were better preserved when stored at a lower SA:V ratio. They concluded that the collected sample should be as large as possible, which minimized background emissions of contaminants and VOC losses; however, both for testing and cleaning, a bag should not be filled more than 80% of its total volume. Similarly, Kasper et al. [76] tested storage properties of several bags of different sizes and materials. They revealed that the material of a bag significantly determined the recovery efficiency of the selected odorants, and they determined the highest degree of recovery for PTFE and Tedlar bags. The selected odorants were also more efficiently preserved in larger bags; the yield was 20% higher for compounds recovered from 10 L Tedlar bag as compared with a 1 L Tedlar bag (63.5% and 42.8%, respectively), which confirmed that higher sample volume/bag surface area favored sample preservation.

### 5.2. Water Vapor Alters Breath Composition during Storage

It has been confirmed that the water vapor content falls rapidly during extended storage, which suggests that sample loss is associated with its diffusion through the bag walls [48]. Since humidity easily permeates through bag walls and adsorbs water-soluble compounds, it constitutes another substantial factor that affects sample stability and recovery [77]. In practice, the initial dry sample tends to reach equilibrium with the humidity level of ambient air, which is more efficient for smaller bags. Hydronium water cluster H_3_O +⋅H_2_O (*m/z* 37) is a characteristic of sample humidity and is commonly employed to assess the water content in a sample with the use of PTR-MS (proton-transfer reaction mass spectrometry) [78]. One should keep in mind that if VOC concentrations are higher in ambient room air, compounds easily diffuse into a bag. Beauchamp et al. [48] confirmed that, within 18 h, the levels of monitored compounds gradually increased. This should be taken into consideration at the stage of designing an experiment to avoid the detection of false-positive signals derived from ambient air. Usually, such an approach requires measuring a blank sample that excludes background contaminants in the actual exhaled breath sample.

### 5.3. Compounds Emitted by Tedlar Bags

Gaseous samples can also be contaminated by compounds released from the bag. Tedlar bags (especially blackened Tedlar bags) exhibit significant emissions of COS and CS2. However, Sulyok et al. [79] compared the suitability of transparent and black-layered Tedlar bags for sulphur compound storage and revealed that standard Tedlar bags guaranteed outstanding stability and recovery of, for example, methylmercaptan. After 7 days, only 10% of the sample was not recovered, while for the black-layered bags, the same depletion was observed after two days. It should also be noted that, due to the specificity of the manufacturing process, Tedlar bags release a certain concentration of phenol (MW = 94) and N-,N-dimethylacetamide (MW = 87) [80]. A more detailed experiment regarding contaminants emitted by Tedlar bag was performed by Mochalski et al. [50]. They determined the concentration of chemical impurities released from a Tedlar bag by measuring representative samples at certain time periods (0, 6, 12, and 24 h after filling). The results revealed that Tedlar bags emitted only nine background compounds: (i) previously mentioned dimethylacetamide, phenol, COS, and CS_2_; (ii) hydrocarbons, such as n-hexane, 2,4-dimethylheptane, and 4-methyloctane; and (iii) acetonitrile (for which the highest concentration was reported) and 1-methoxy-2-propylacetate. It should be mentioned that some compounds were also detected in ambient air, which confirmed the possibility of their permeation through bag walls.

### 5.4. Temperature Affects the Levels of VOCs in Exhaled Breath

Li et al. [81] determined how storage temperature affects the stability of breath VOCs in Tedlar bags. The collected breath samples were spiked with the selected carbonyl VOCs, divided into aliquots, and stored at 4 °C or room temperature. The recovery level of carbonyl VOCs was higher for those samples stored at a lower temperature and, to avoid significant sample loss, storage time should not exceed 2 h.

It should be noted that the structure of polyvinyl fluoride is highly irregular, which fosters adsorption of compounds. This process is also facilitated by adsorbate properties, such as (i) the ability to form hydrogen bonds; (ii) its polarity, and (iii) capability for van der Waal’s interactions. Moreover, less volatile compounds with lower vapor pressure are more easily adsorbed on polymer film, while those with higher vapor pressure exhibit insignificant adsorption rates [52].

## 6. Sample Enrichment

### 6.1. Thermal Desorption Tubes as Sample Preconcentrators

As previously mentioned, sampling with the use of Tedlar bags does not separate breath fraction which may have a slight impact on VOC concentrations in exhaled breath. Preconcentration methods can detect VOCs that occur in exhaled breath at trace concentrations. Sorbent-containing tubes [82], solid phase microextraction [83], and needle trap devices [84] can be employed to concentrate analytes. Among them, thermal desorption (TD) tubes are most frequently used in laboratory practice and can be easily adapted to various compounds [46]. Such enrichment devices consist of stainless-steel or glass tubes that contain the adsorbent. According to Marce et al. [85], an ideal sorbent should: (i) be inexpensive and easy-to-use, (ii) have infinite breakthrough volume for the breath constituents, (iii) enable complete desorption at moderate conditions, and (iv) not generate any artefacts. Tenax TA is one of the commonly used porous polymer (based on the 2,6-diphenylene oxide) that can be employed as the sorbent material in a breath assay. Because of its specific properties, it is intended to be used for capturing heavier and less volatile compounds (C6–C30) [86]. To extend the range of constituents that can be determined, one should use two- or multibed sorbent tubes, however, for many purposes, the sampling range guaranteed by Tenax TA TD tubes is adequate. The employment of multibed tubes requires that the sorbents are grouped in order from the weakest to the strongest, which prevents premature desorption of more volatile compounds and precludes irreversible retaining of less volatile species in the strongest sorbent [87]. Despite the relatively low surface area (35 m^2^/g), Tenax TA seems to be the best candidate among other available adsorbents, especially in experiments in which water-derived analytical issues should be avoided. Because of low affinity for water, Tenax TA is also useful for capturing VOCs from humid samples [49]. Selected types of adsorbents and their properties are given in Table 4.

### 6.2. TD Tubes Conditioning

Before each use, a TD tube must be conditioned. This procedure is usually determined by the manufacturer and involves heating the tube above its desorption temperature in a steam of inert, high purity gas. Several options for tube conditioning are listed below in Table 5.

### 6.3. Breath Sampling onto TD Tubes

VOC preconcentrations can be performed actively or passively. Passive sampling involves a free diffusion of molecules from a sample to a collecting agent, while active sampling involves pumping a sample through a sorbent bed. It is worth noting that the same sorbents can be employed both for active and passive sampling [85]. In order to perform active sampling, breath collected, for example, in a Tedlar bag, should be transferred onto sorbent tubes. In practice, it involves the use of a pump which deflates the bag with subsequent transfer of the sample through a sorbent. Several options for active sampling are depicted in Table 6.

During enrichment, it is essential to use a tube in a vertical position which counteracts undersampling, and the tube should be capped at both ends immediately after sampling. Additionally, a drying stage can be introduced to remove traces of water [82]. There are four main options to overcome the water-vapor issue: (i) pass the sample through a drying agent; (ii) dry the sample using high-purity inert gas; (iii) warm the sorbent during enrichment; or (iv) reduce the sample volume, which is only possible when single/small breath samples have been taken. Regardless of the approach chosen, one should keep in mind that drying has its drawbacks such as sample loss and/or its contamination [97].

### 6.4. Compounds Emitted by Tenax TA Tubes

It should be noted that Tenax TA exhibits heat-induced depolymerization during which benzene, styrene, benzaldehyde, acetophenone, octanal, nonanal, decanal, 2,6-diphenylquinone, and 2,6-diphenylhydroquinone can be released [98]. Furthermore, various artefacts can be generated as a consequence of sorbent exposure to O_3_, NO_2_, or sample degradation, which may cause false-positive detection of breath constituents; however, any other temperature sensitive adsorbent may show some level of background contamination [99].

### 6.5. Sample Storage and Stability of TD Tubes

Usually, trapping of VOCs is not immediately proceeded with TD-GC-MS analysis, because breath samples cannot be taken from all patients at the same time. Preconcentration must be conducted within due time after collecting, which significantly reduces the number of samples that can be taken per day. Moreover, not all research units own suitable analytical instruments, and therefore, samples must be sent to off-site facilities. In either case, sorbent tubes must ensure adequate sample storage and stability; it is commonly known that relative humidity (RH), temperature (T), and sampling flow rate (FR) impact these two features, and therefore, have been tested in several experiments. It has been revealed that particular compounds (toluene, benzene, and xylene) loaded in a dry atmosphere were stable during storage even for 25 months [98]. In turn, Huang et al. [100] determined the influence of RH on the trapping of aliphatic hydrocarbons using multibed (Tenax TA, Crabograph 1TD, and Carboxen 1003) sorbent tubes. They confirmed that atmospheric water had an effect on breakthrough volume, which significantly decreased sampling efficiency. Moreover, introducing a dry-purging step did not improve the obtained results and led to a loss of several volatile analytes. Brown et al. [90] compared the stability of nine chemicals (n-hexane, 4-methylpentan-2-one, toluene, n-butyl acetate, cyclohexanone, 1,2,3-trimethylbenzene, phenol, 4-phenylcyclohexanone, and n-hexadecane) loaded onto multi-sorbent tubes in an atmosphere of 0% and 40% relative humidity, and determined that the storage performances of these tubes were equal to those of tubes packed with Tenax TA, however, the storage time should not exceed 4 weeks. They confirmed that the single sorbent Tenax TA TD tube could be successfully employed in VOC analysis [89].

## 7. Exhaled Breath Analysis Using Thermo-Desorption Gas Chromatography Mass Spectrometry (TD-GC-MS)

### 7.1. Thermal Desorption

Thermal desorption (TD) is a technique that employs sorbent-containing devices to concentrate VOCs prior to injection for gas chromatography. In principle, heating the sorbent increases the volatility of captured compounds which are then swept by a flow of inert gas into a cooled-below-RT secondary (focusing) trap. In the next stage, the secondary trap is heated and the VOCs are transferred (with the reverse flow of carrier gas) to a gas chromatography coupled with mass spectrometry instrument for separation and analysis [100]. Primary desorption is usually carried out at about 250–350 °C and the applied temperature should be lower than the temperature limit of the particular sorbent. The secondary trap is cooled by liquid nitrogen or carbon dioxide and operated with the temperature ranging from −150 to 25 °C (for details see Table S1). It may contain various types of sorbent materials, such as: Tenax TA, Tenax GR, T12ME-2S, Carbograph 1, Carbograph 5TD, and Carbosieve S-III. The particular adsorbent together with low temperature is intended to minimize band broadening and to improve the detection limit as well as the efficiency of the GC-MS analysis and separation. The temperature of secondary desorption is usually lower than the temperature of primary desorption (ΔT ranging from −30 °C to +50 °C) (see Table S1). Volatile compounds released from the TD unit are transferred through a heated transfer line to a GC column and because each molecule interacts differently with the stationary phase, breath components can be separated. To improve the peak shapes, resolution, and accelerate the elution of compounds with higher boiling points, a temperature gradient should be applied.

### 7.2. Gas Chromatography Mass Spectrometry

In mass spectrometry, compounds are ionized and separated according to their mass-to-charge ratios (*m/z*) [101]. This analytical method enables data to be acquired in two ways: (i) scan over the mass range to acquire a library-searchable full-scan mass spectra of interest and (ii) single/selected ion monitoring scan to detect, at much higher sensitivity, the compounds selected masses of interest [102]. The first scan mode (full scan mode) is generally utilized for untargeted breath analysis, which enables the identification of all relevant compounds and the detection of a prospective biomarker. The second mode (selected ion monitoring mode (SIM)) is rather suitable for targeted analysis which aims to measure a predefined chemical entity [103].

Samples stored in TD tubes can be analyzed on any high-resolution mass spectrometer, including a GC-Q-TOF (gas chromatography quadrupole time-of-flight mass spectrometer) and a GC orbitrap spectrometer. Moreover, the TD frontend can be coupled to a PTR-TOF-MS (proton-transfer reaction time-of-flight mass spectrometry) system, which has been employed in an experiment where the ReCIVA device was used as a sample collector. Using PTR, such an approach is more suitable for real-time identification, which does not require a compound separation step [104]. More recently, Monks et al., for the first time, employed two-dimensional gas chromatography (GC x GC) with dual flame ionization and mass spectrometric detection to analyze VOCs. They developed a method that utilized a ReCIVA device and automated thermal desorption to improve chromatographic separation and to enable a continuous analysis of breath and environmental samples [105].

With the use of GC-MS, breath samples can be precisely analyzed and a wealth of information regarding sample composition can be gathered. Moreover, the identity of particular compounds can be confirmed against spectral databases, such as the National Institute of Standards and Technology (NIST), the Human Metabolome Database (HMDB), and the Human Breathomics Database (HBDB), which also provide information about the compounds’ physical properties, spectral profiles, biological activities, and metabolic pathways [106]. Although TD–GC–MS is widely considered to be the gold standard analytical method for off-line breath investigations [107], it is not commonly used in clinical practice, mainly due to a lack of reproducible protocols for analyses, inappropriate data processing methods, and also instrument variability issues [108]. Especially, time-dependent instrument variability poses a challenge in every analysis, since it is common that the results may vary for the same concentration of a sample analyzed on different days. Similarly, different instruments (types of mass analyzers) may output misleading results. Wang et al. [109] determined that a GC-QTOF-MS instrument exhibited a lower variation in the measured values than a single-quadrupole GC-MS instrument, both within the same day and over a four-week period of analysis. However, the first measurement obtained with the use of GC-QTOF-MS varied from all of the other quantifications, which determined the need to normalize each dataset.

### 7.3. Quality Assessment

It is also recommended to use a set of samples that controls and monitors the separations, which includes running an empty tube and a standard mix. Moreover, each sample should be spiked with one or more internal standards that differ in their retention time, which enables data normalization [99]. An internal standard addition can only be skipped when the compounds emitted from the matrix are unknown [110]. To monitor the quality of the TD-GC-MS analysis as well as exclude background contamination, (i) adsorbent (due to its thermal instability), (ii) adsorbent purged with clean nitrogen or helium (depending on the one used in the experiment), (iii) adsorbent spiked with internal standard, (iv) high purity grade gas transferred from a clean bag (to monitor compounds released from sampling container), (v) standard VOC mixture, and (vi) quality control samples (injected at regular intervals) should also be included in the TD-GC-MS worklist [111]. It has to be emphasized that all chemical standards are used to calibrate the TD-GC-MS system and to confirm the retention time and/or concentration of detected compounds [50]. Liquid standards should be: (i) of high purity; (ii) more volatile than the analytes of interest, and (iii) not prepared in solvents that can be retained by the adsorbents [112]. Generally, in air analysis, standards can be injected directly onto sorbents using an unheated GC injector, syringe or automatically applied by thermal desorber [113,114]. Commonly used internal standards include: bromochloromethne, p-bromofluorobenzene, chlorobenzene-d5, 1,4-difluorobenzene, trichlorofluoromethane, tolunene-d8, decane-d22, and hexadecane-d34 [45,90,112,113,114,115,116].

Breath analysis poses a challenge to obtaining consistent and reproducible results. One of the utmost important strategies to minimize false positive identification is the analysis of blanks. To avoid undesired bias, contaminants should be identified and removed a priori from the final data matrix, before further data treatment. Therefore, the incorporation of blanks should be treated as an obligatory element in study design. Best practices for breath VOC analysis include consideration of contamination originating from Tedlar bags, air, and the analytical platform. The chemical background substantially rises due to the presence of N,N-dimethylacetamide (DMAC) and phenol emitted by the Tedlar bags. Therefore, blank trapping such contaminants prepared from the bag filled with pure nitrogen should be included. In addition, system blanks can identify instrument artefacts such as polydimethylsiloxanes. Furthermore, an air blank (laboratory and sampling room) employed prior to each analytical batch is obligatory to reduce systematic and random variation from sampling and analysis.

## 8. Conclusions

VOCs in exhaled breath can be easily detected using off-line methods where thermal desorption tubes and gas chromatography coupled to mass spectrometry are considered to be the gold standard. So far, nearly 200 compounds have been successfully identified, however, many of them may still have an exogenous origin [117]. Moreover, VOCs can be found in skin emanations, urine, saliva, blood, and feces [118,119,120,121,122]. For those compounds that have been well-characterized and appear only in exhaled breath, it is essential to determine their biochemical origin. For that purpose, it is crucial to develop a standardized method of breath sampling and analysis, which may find application in clinical practice in the near future. This review provides a snapshot of the most common approaches used for the analysis of volatile organic compounds in breath samples. The number of studies on breath composition is rapidly growing; however, there are many obstacles and important shortcomings that should be taken into consideration from the very first beginning of the conducted projects. The overall study design with all aspects of robust and reproducible procedures applied is essential to produce clinically meaningful results. Herein, for such a specific sample type, the preanalytical phase can have a major impact on the downstream analysis. Therefore, recognizing the possible factors associated with unwanted bias and disseminating the quality assessment procedures are the best strategies to minimize the risk of false discoveries.

## Figures and Tables

**Table 1:** Tedlar bag’s cleaning guidelines.

	Purge		Heating		
Bag	Flushing Agent	Rep	Temp.	Duration (min)	Ref.
1.0 L, transparent	Synthetic air	1	60 °C	720	49
1.0 L, black-layered	Synthetic air	1	60 °C	<120	40
3.0 L, transparent	Synthetic air	3	45 °C	15	48
		1	95 °C	600	
3.0 L, transparent	Synthetic air	3	50 °C	n/a	51
3.0 L, transparent	High-purity N_2_	5	n/a	n/a	50
		1	95 °C	600	
10.0 L, transparent	High-purity N_2_	5	Up to 27 °C	n/a	52

**Table 2:** Details regarding off-line breath sampling.

Bag Volume (L)	Mode of Exhalation	Disease	Age	Ref.
1	No special provisions	Asthma	Children	[20]
3	No special provisions	n/a	Adults	[63]
3	No special provisions	Irritable bowel syndrome	Adults	[64]
4	Take a deep breath Hold for 4 s Exhale smoothly	Hepatocellular carcinoma	Adults	[65]
5	No special provisions	Asthma	Children	[66]
5	Inhale deeply Exhale slowly	Asthma	Children	[67]
5	Deep inhale Exhale into resistance free Tedlar bag	n/a	Adults	[62]
5	Inhale Hold for 5 s Fully exhale	Asthma	Adults	[68]
5	No special provisions	Non-alcoholic steatohepapatitis	Adults	[69]
5	Inhale Hold for 5 s Exhale	n/a	Adults	[70]
5	Breath at normal rate	Sarcoidosis	Adults	[71]
5	Bag was tightly connected to the limb of the ventilator	Pneumonia	Adults	[72]
5	No special provisions	Liver cirrhosis	Adults	[73]
10	No special provisions	Asthma	Adults	[74]

**Table 3:** The SA:V ratios that correspond to particular sample sizes analyzed in Mochalski et al. study.

Volume (L)	Capacity (%)	SA:V (m-1)
0.6	20%	291
1.2	40%	145
2.4	80%	73

**Table 4:** Selected types of adsorbents and their properties.

Adsorbent Type	Sampling Range	T Max (°C)	Surface Area Range (m^2^/g)	Water Content (mL/g) 20 °C
Porous organic polymers (e.g., Tenax TA, GR, chromosorb)	C1–C30	250–350	35–170	40–180
Carbon-based materials (e.g., carboxen)	C2–C5	>400	400–1200	200–800
Graphitized carbon black (e.g., carbotrap)	C3–C20	>400	5–560	N/A

**Table 5:** Commonly employed conditions for thermal desorption tube conditioning.

Gas	Gas Purity	Flow Rate (mL/min)	Temperature (°C)	Time (min)	Ref.
Nitrogen	99.999%	85	320	60	[45]
Helium	99.999%	50	330	30	[51]
Helium	n/a	50	(1) 300 (2) 325	(1) 30 (2) 120	[88]
Nitrogen	n/a	75	320	600	[89]
Helium	n/a	60	(1) 320 (2) 335	(1) 60 (2) 30	[90]
Nitrogen	n/a	100	335	30	[91]

**Table 6:** Conditions of commonly used active sampling methods.

Adsorbent	Flow Rate (mL/min)	Sampling Pump	Ref.
Tenax TA	100	Gilian^®^ GilAir^®^ PLUS	[42]
Tenax TA	200	Gilian Gil Plus Pump	[45]
Tenax TA	200	ACTI-VOC, Markes	[91]
Tenax TA	270	MultiRAE Pro	[92]
Tenax GR	50	n/a	[53]
Tenax GR	250	peristaltic pump	[75]
Tenax GR	250	peristaltic pump	[93]
Tenax TA/Carbograph 1TD	22	ACTI-VOC, Markes	[94]
Tenax TA/Carbograph 5	200	Pocket Pump SKC	[51]
ORBOTM 420 and Tenax TA	100	Schego membrane pump	[95]
CarboxenTM 1003, CarbopackTM B and CarbopackTM Y	25	n/a	[96]

## Supplementary Materials

The following supporting information can be downloaded at https://www.mdpi.com/article/10.3390/metabo13010008/s1, Table S1: Commonly employed TD-GC-MS conditions for breath constituent analysis.
