# Supplementary material for: Common Strategies and Factors Affecting Off-Line Breath Sampling and Volatile Organic Compounds Analysis Using Thermal Desorption-Gas Chromatography-Mass Spectrometry (TD-GC-MS)
Source: Metabolites. 2022 Dec 21;13(1):8. doi: 10.3390/metabo13010008 (PMC9866406; doi:10.3390/metabo13010008)
Supplement: Supplementary file 1 [file metabolites-13-00008-s001.zip › metabolites-2007387-supplementary.pdf]

Thermal desorption				GC-MS analysis				
**Sorbent**	**Thermal desorber**	**Cold trap**	**Conditions**	**GC-MS**	**Column**	**GC separation**	**MS parameters**	**Ref.**
TenaxTA, Quartz wool/TenaxTA /Carbograph TM1 5TD, Quartz wool/TenaxTA /Carbopack TM2	Markes TD-100TM	U-T12ME-2S containing quartz wool, TenaxTA and and Carbograph 5TD	**Pre-purge:** 1 min. at 30 mL/min. **Primary desorption:** 8 min. at 280 °C, 50 mL/min. He **Cold trap low:** - 10 °C **Cold trap high:** 3 min. at 300 °C	Agilent 6890 GC and 5973 mass spectrometer	DB5 (60 m x 0.25 mm x 0.5 μm)	f = 1.3 mL/min. 35 °C – 1 min. 2 °C/min. to 75 °C 5 °C/min. to 140 °C 10 °C/min. to 250 °C 5 min. hold	*m/z* 20-450 MS source temperature: 250 °C MS quad temperature: 150 °C	V. M. Brown et al. J. Chromatogr. A, 1350, (2014), 1-9.
Tenax TA	UNITY	Cold trap designed for sulphur compounds packed with graphitized carbon black	**Primary desorption:** 10 min. at 280 °C, 20 mL/min. He **Cold trap low:** - 10 °C **Cold trap high:** 280 °C	Agilent 6890 GC and 5975 mass spectrometer	DB1 60 m x 0.32 mm x 5 μm	60 °C – 7 min. 15 °C/min. to 230 °C 230 °C – 5 min.	*m/z* 33 - 100	P. Mochalski et al. J. Chromatogr. B, 877, (2009), 1856-1866.
Tenax TA, Tenax/Carbograph 5TD	Markes Internation al TD-100xr	International Air Toxics	**Pre-purge:** 1 min. at 20 mL/min. He **Primary desorption:** 10 min. at 310 °C **Cold trap high:** 315 °C, heating rate 40 °C/s for 5 min.	Trace Ultra-ISQ GC-MS system (Thermo Scientific)	Rxi-624Sil 60 m x 0.32 mm x 1.8 μm	f = 2 mL/min. 40 °C – 1 min. 10 °C/min. to 240 °C 240 °C – 20 min.	EI = 70 eV *m/z* 35-300 with 0.154 spectra/s	S.W. Harshman et al. J. Breath Res., 14, (2020), 016009.
TenaxGR	STD 1000	Internal focusing trap packed with TenaxGR	**Primary desorption:** 5 min. at 250 °C, 35 mL/min. He **Cold trap low:** 5 °C **Cold trap high:** 250 °C	Trace GC Ultra GC and Trace DSQ quadrupole mass spectrometer (Thermo Electron Corporation)	DB-624 60 m x 0.25 mm x 1.4 μm (6% cyanopropyl phenyl siloxane and 94%	35 °C – 10 min. 4 °C/min. to 130 °C 130 °C – 2 min. 20 °C/min. to 250 °C 250 °C – 10 min. 25 °C/min. to 260 °C 260 °C – 15 min.	EI = 70 eV *m/z* 18 - 200	S. Ghimenti et al. J. Breath Res., 9, (2015), 047110.

					dimethylpolysiloxane)			
Tenax TA, Tenax TA/Carbograph 5TD	Markes TD-100xr	Air Toxics	**Pre-purge:** 1 min. at 20 mL/min., N_2 **Primary desorption:** 10 min. at 310 °C, 50 mL/min. He **Cold trap high:** 5 min. at 315 °C, heating rate 40 °C/s	Trace Ultra-ISQ (Waltham)	Rxi-624Sil (60 m × 0.32 mm × 1.8 µm)	f = 2 mL/min. 20 °C – 1 min. 10 °C/min. to 240 °C 240 °C – 20 min.	EI = 70 eV MS source temperature: 275 °C *m/z* 35 – 300 (0.154 s/scan)	S. W. Harshman et al. J. Breath Res., 14, (2020), 036004.
Tenax TA	Automated Thermo Desorber	Cold trap packed with TenaxTA	**Primary desorption:** 5 min. at 250 °C, 7,2 mL/min. He **Cold trap low:** -10 **Cold trap high:** 250 °C	-	-	-	-	C. Merlen et al. Microchem. J., 132, (2017), 143-153.
Tenax® TA, Tenax® TA/Carbopack™, Tenax® TA/Sulficarb, Tenax® TA/ Carbograph™ 5TD, Tenax® TA/Carbograph™ 1TD/Carboxen® 1003, Carboxen® 1016/Carbograph™ 5TD	TD100-xr thermal desorber	N/A	**Pre-purge:** 3 min. at 20 mL/min. **Primary desorption:** 5 min. at 300 °C, 50 mL/min. **Cold trap low:** -10 °C **Cold trap high:** 5 min. at 300 °C,	Pegasus 4D HRT (LECO Corporation) GC ×GC-HR ToF MS instrument with an Agilent 7890 GC	**First dimension:** Rxi-624SilMS (30 m × 0.25 µm × 1.4 µm) **Second dimension:** Stabilwax (2 m × 0.25 µm × 0.5 µm)	f = 1 mL/min. 45 °C – 3 min. 5 °C/min. to 240 °C 240 °C – 5 min.	MS source temperature: 250 °C *m/z* 29-450	F. Franchina et al. Talanta, 222, (2021), 121569
TenaxGR, TA1TD, TA5TD	TD-100	General purpose hydrophobic trap (Markes International)	**Pre-purge:** 2 min. at 50 mL/min., He **Cold trap low:** 0 °C **Desorption:** 330°C	7890B GC, Agilent and triple-quadrupole mass	DB-5 (30 m x 0.25 mm x 25 µm)	f = 1.3 mL/min. 40 °C – 0 min. 6 °C/min. to 170 °C 170 °C – 0 min.	EI = 70 eV *m/z* 40-500	M. Wilkinson et al. J. Breath Res., 14, (2020), 46006.

				spectrometer 7010, Agilent		15 °C/min. to 190 °C		
Tenax GR	Gerstel TDS	Trap packed with Tenax TA	**Primary desorption:** 3 min. at 300 °C<br>**Cold trap low:** - 150 °C<br>**Cold trap high:** 2 min. at 280 °C, heating rate 20 °C/s.	7890 N GC, Agilent and time-of-flight mass spectrometer, LECO Pegasus	VF1-MS column (30 m × 0.25 mm, 1 μm)	f= 1.2 mL/min.<br>40 °C – 5 min.<br>10 °C/min. to 300 °C<br>300 °C – 5 min.	EI = 70 eV<br>*m/z* 29-400	P. Brinkman et al. Eur. Respir. J., 55, (2020), 1900544.
Carbopack Y/X/Carboxen 1000, Carbopack Y, Tenax TA, Carbopack B/X	Gerstel thermal desorption unit		**Primary desorption:** 2 min. at 20 °C followed by heating to 300 °C (for TenaxTA) or 330 °C (for other sorbents)<br>**Cold trap low:** - 30 °C<br>**Cold trap high:** 330 °C, heating rate 12 °C/sec	Pegasus 4D LECO Corporation GC x GC-TOF MS instrument with an Agilent 7890 GC	**First dimension:** Rxi-624Sil (60 m x 250 mm x 1.4 μm)<br>**Second dimension:** Stabilwax (1.3 m x 250 mm x 0.5 mm)	f = 2 mL/min.<br>45 °C – 1 min.<br>8 °C/min. to 235 °C	*m/z* 40-400 (150 scans/s)<br>MS source temperature: 200 °C	F. Franchina et al. Anal. Chim. Acta, 1066, (2019), 146-153.
Tenax GR	Automated thermal desorber, Gerstel	Cold trap packed with Tenax TA	**Primary desorption:** 3 min. at 300 °C<br>**Cold trap low:** - 150 °C<br>**Cold trap high:** 280 °C, heating rate 20 °C/s	GC 7890 N, Agilent and time-of-flight mass analyser, LECO Pegasus 4D, LECO.	VF1-ms, 30 m × 0.25 mm, 1 μm	f = 1.2 mL/min.<br>40 °C – 5 min.<br>10 °C/min. to 300 °C<br>300 °C – 5 min.	EI = 70 eV<br>*m/z* 29-450	W. M. Ahmed et al. J. Breath Res., 13, (2018), 016001.
Tenax/Carbograph-5TD	MD-100, Markes	T12ME-2S	**Pre-purge:** 3 min. at 50 mL/min., He<br>**Primary desorption:** 10 min. at 280 °C<br>**Cold trap low:** 10 °C<br>**Cold trap high:** 290 °C, heating rate 99 °C/min.	Agilent 7890 GC and 5977A MSD mass spectrometer, Agilent	ZB-642 capillary column (60m x 0.25mm ID x 1.40 μm)	f = 1.0 mL/min.<br>40 °C – 4 min.<br>5 °C/min. to 100 °C<br>100 °C – 1 min.<br>5 °C/min. to 110 °C<br>110 °C – 1 min.<br>5 °C/min. to 200 °C<br>200 °C – 1 min.	EI = 70 eV<br>MS source temperature: 230 °C<br>*m/z* 20 – 250 (6 scans/s)	G. B. Hannah et al. J. Breath Res., 12, (2017), 016007.

						10 °C/min. to 240 °C<br>240 °C – 4 min.		
Carbograph 1TD/Carbopack X	Markes Unity desorption unit, Markes	n/a	**Primary desorption:** 350 °C<br>**Cold trap low:** 5 °C<br>**Cold trap high:** 300 °C	GC Ultra, Thermo Electron Corporation, , Thermo Electron Tempus Plus time-of-flight mass spectrometer, Thermo Electron Cormporation	RTX05ms (30 m x 0.24 mm x 1 µm)	40 °C – 5 min.<br>10 °C/min. to 270 °C<br>270 °C – 5 min.	EI = 70 eV<br>*m/z* 35 – 350 (5 scans/s)	R. R. Fijten et al. J. Breath Res., 12, (2017), 016004.
Tenax TA	Markes Internation al TD-100	Air Toxics packed with Carbograph 1 and Carbosieve S-III	**Primary desorption:** 10 min. at 310 °C<br>**Cold trap low:** 25 °C<br>**Cold trap high:** 315 °C, heating rate 40 °C/s	Trace Ultra-ISQ GC, Thermo Scientific and single quadrupole mass spectrometer, Waltham)	Rxi-624Sil (60 m x 0.32 mm x 1.8 µm)	f = 2.0 mL/min.<br>40 °C – 0 min.<br>10 °C/min. to 240 °C<br>240 °C – 20 min.	EI = 70 eV<br>MS source temperature: 275 °C<br>*m/z* 35 – 300<br>0.154 s/scan	S. W. Harshman et al. J. Breath Res., 10, (2016), 046008.
Tenax TA/ Carbograph 1 TD	Markes Unity Series 1 thermal desorption unit	A general purpose hydrophobic cold trap	**Desorption:** 5 min. at 300 °C<br>**Cold trap low:** - 10 °C<br>**Cold trap high:** 300 °C, heating rate max °C/min.	Varian 3800 GC and Varian Saturn 4000 ion-trap mass spectrometer,	DB-5 (60 m x 0.25 mm x 0.25 µm)	f = 2.0 mL/min.<br>40 °C – 0 min.<br>5 °C/min. to 300 °C<br>300 °C – 8 min.	*m/z* 40 – 445<br>0.38 s/scan	S. Kang et al. J. Breath Res., 10, (2016), 026011.
1. Carbograph 2TD/1TD,	n/a	n/a	**Desorption:**<br>**1,2,3 and 6**: 20 min. at 375 °C<br>**4,5:** 15 min. at 280 °C	PerkinElmer (PE) 650 TurboMatrix	Rxi-5Sil MS capillary GC column with a 5 m	f = 2.0 mL/min.<br>35 °C – 2 min.<br>6 °C/min. to 190 °C	MS source temperature: 290 °C<br>*m/z* 35 – 300	M. A. G. Wallace et al. J. Chromatogr. A,

2. Quartz wool-Carbograph 2 TD,<br>3. Carbograph 2TD,<br>4. Tenax TA,<br>5. PAH,<br>6. XRO-440			**Cold trap low:** 10 °C<br>**Cold trap high:** 385 °C, with 10 min. hold	ATD system and an Agilent 6890 N GC coupled to an Agilent 5975 inert XL MS	Integra Guard column, 30 m x 0.25 mm ID, 0.25 µm	28 °C/min. to 310 °C<br>310 °C – 8 min.		1602, (2019), 19-29.
1. Chromosorb 106,<br>2. Tenax TA<br>3. Porapak N,<br>4. Tenax GR/ Carbopack B, Carbosieve SIII	TD2 (Chromatec, Russia)	n/a	**Desorption:**<br>5 min. at<br>1. 220 °C<br>2. 250 °C<br>3. 150 °C<br>4. 250 °C<br>**Cold trap low:** -10 °C<br>**Cold trap high:**<br>1. 220 °C<br>2. 250 °C<br>3. 150 °C<br>4. 250 °C<br>Hold for 2 min.	Chromatec crystal 5000.2, coupled with a quadrupole mass spectrometer (Chromatec MSD)	Supelco Supel-Q PLOT (30 m x 0.32 mm)	f = 1.3 mL/min.<br>50 °C – 0 min.<br>10 °C/min. to 150 °C<br>6 °C/min. to 220 °C<br>250 °C – 4 min.	EI = 70 eV<br>MS source temperature: 200 °C<br>*m/z* 29 – 250	E. Gashimova et al. Heliyon, 6, (2020), e04224.
Tenax TA/Carbograph 5 TD	TDUnity 2 (Markes International Ltd.)	U-T4WMT-2S (Markes International Ltd.)	**Pre-purge:** 3 min. at 5 mL/min., He<br>**Desorption:** 10 min. at 300 °C<br>**Cold trap low:** 20 °C<br>**Cold trap high:** 300 °C<br>**Desorption flow:** 30 mL/min.	GC-Agilent 7890 and a mass spectrometer MS-Agilent 5975 (Agilent Technologies)	VOCOL® diphenyl dimethyl polysiloxane (60 m × 0.25 mm ID, 1.5 µm) (Supelco)	f = 1.7 mL/min.<br>37 °C – 5 min.<br>6 °C/min. to 190 °C<br>2 °C/min. to 200 °C<br>15 °C/min. to 220 °C<br>220 °C – 3 min.	n/a	A. Di Gilio et al. Cancers, 12, (2020), 1262.
Carbotrap 200, Carbotrap, Carbosieved SIII	TD Unit ACEM 900 (CDS,Quad Services)	n/a	**Desorption:** 5 min.. at 350 °C<br>**Cold trap low:** 35 °C<br>**Cold trap high:** 350 °C	GC 8000 TOP coupled with mass spectrometric detector	n/a	f = 1.3 mL/min.<br>35 °C – 5 min.<br>6 °C/min. to 190 °C	n/a	E. Postaire et al. Ann. Pharm. Fr., Elsevier Masson, 2020, p. 34-41.

				device Automass II (electron ionization) (Finnigan Corporation, Quad Services)		2 °C/min. to 200 °C<br>15 °C/min. to 220 °C<br>220 °C – 3 min.		
Tenax GR/Carbopack B	TD100-xr (Markes International Ltd.)	n/a	**Pre-purge:** 3 min. at 20 mL/min.<br>**Desorption:** 5 min. at 290 °C<br>**Cold trap low:** - 10 °C<br>**Cold trap high:** 300 °C	Pegasus GCHRT 4D (LECO Corporation) with an Agilent 7890 GC	**First dimension:** Rxi-624SilMS (30 m × 0.25 mm × 1.4 µm) **Second dimension:** Stabilwax (2 m × 0.25 mm × 0.5 µm) (Restek Corporation)	f = 1.0 mL/min.<br>35 °C – 5 min.<br>5 °C/min. to 240 °C<br>240 °C – 5 min.<br>Secondary oven: +5 °C and +15 °C for the quad-jet dual-stage thermal modulator	MS source temperature: 250 °C<br>*m/z* 29 – 450	D. Zanella et al. Anal. Bioanal. Chem., 413, (2021), 3813-3822.
Tenax TA/carbograph 5TD	TD-100 (Markes International)	U-T12ME-2S, Material/Emission, C4-C32 (Markes international)	**Desorption:** 3 min. at 300 °C<br>**Cold trap low:** 20 °C<br>**Cold trap high:** 300 °C	Q Exactive GC Hybrid Quadrupole-Orbitrap Mass Spectrometer (Thermo Fisher Scientific)	TraceGOLD TG-624SilMS; (Thermo Fisher Scientific)	40 °C – 1 min.<br>10 °C/min. to 270 °C<br>30 °C/min. – 300 °C<br>300 °C – 5 min.	EI = 70 eV<br>MS source temperature: 230 °C<br>*m/z* 30 – 450	G. Ferrandino et al. Clin. Transl. Gastroenterol., 11, (2020), 1-5.
Carbograph 1TD	TD-100 xr (Markes International Ltd.)	Hydrophobic, general purpose (Markes International Ltd.)	**Pre-purge:** 1 min. at 50 mL/min.<br>**Desorption:** 5 min. at 300 °C<br>**Cold trap low:** - 10 °C<br>**Cold trap high:** 300 °C	Agilent 7820A with 5977B MS (Agilent Technologies Ltd.)	DB-5MS (60 m×0.25 mm×0.25 µm) (Agilent	f = 1.0 mL/min.<br>35 °C – 0 min.<br>2.8 °C/min. to 130 °C<br>4 °C/min. to 220 °C	MS source temperature: 230 °C<br>*m/z* 40 – 350	W. Ibrahim et al., ERJ Open Res., 7, (2021), 00139.

					Technologies Ltd.)	25 °C/min. to 320 °C<br>320 °C – 10 min.		
TenaxGR	TD-100 (Markes International)	U-T11GPC-2S (Markes International)	**Pre-purge:** 1 min. at 50 mL/min.<br>**Desorption:** 5 min. at 280 °C<br>**Cold trap low:** 0 °C<br>**Cold trap high:** 280 °C	Agilent 7890B GC; Agilent 7010 triple quadrupole	DB5 ms GC (0.25 µm, 0.25 mm × 30 m) (Agilent Technologies)	f = 1.0 mL/min.<br>40 °C – 0 min.<br>6 °C/min. to 170 °C<br>15 °C/min. to 250 °C<br>250 °C – 2 min..	EI = 70 eV<br>*m/z* 40 – 500	P. M. van Oort et al., Analyst, 146, (2021), 222-231.
Carbotrap-B, Carbopack-X	TD-100 (Markes International)	n/a	**Desorption:** 10 min. at 320 °C<br>**Cold trap low:** - 20 °C<br>**Cold trap high:** 340 °C	7890B GC (Agilent Technologies) Bench-TOFdx (Five Technologies)	Restek-Q-Bond (30 m x 0.25 mm x 8 µm) (Restek Corporation)	n/a	n/a	A. Pizzini et al. J. Breath Res., 12, (2018), 036002.
